# Hemolytic Uremic Syndrome Following Holmium Laser Enucleation of the Prostate: A Case Report

**DOI:** 10.7759/cureus.85599

**Published:** 2025-06-09

**Authors:** Wojciech Krajewski, Lukasz Nowak, Patryk Patrzałek, Wojciech Tomczak, Joanna Chorbinska, Jan Laszkiewicz, Bartosz Malkiewicz, Tomasz Szydelko

**Affiliations:** 1 University Center of Excellence in Urology, Department of Minimally Invasive and Robotic Urology, Wroclaw Medical University, Wroclaw, POL; 2 University Center of Excellence in Urology, Wroclaw Medical University, Wroclaw, POL

**Keywords:** hemolytic uremic syndrome (hus), holep, holmium laser enucleation of prostate (holep), laser enucleation, prostate hyperplasia

## Abstract

Holmium laser enucleation of the prostate (HoLEP) is a widely used surgical treatment for benign prostatic hyperplasia (BPH), and it is associated with a low incidence of severe complications. We report a rare case of hemolytic uremic syndrome (HUS) following HoLEP in a 71-year-old male. Shortly after the surgery, the patient developed altered consciousness, jaundice, and multiorgan failure, necessitating intensive care and renal replacement therapy. HUS was identified as the underlying cause. After a month-long hospitalization, the patient showed progressive renal recovery. This report underscores the importance of early recognition, multidisciplinary management, and close follow-up in achieving favorable outcomes in rare post-HoLEP complications.

## Introduction

Holmium laser enucleation of the prostate (HoLEP) is a widely recognized and minimally invasive treatment for symptomatic benign prostatic hyperplasia (BPH). The procedure involves the use of a holmium laser to remove obstructive prostate tissue and is a safe operation with low complication rates [1,2]. Hemolytic uremic syndrome (HUS) is a rare, potentially life-threatening condition characterized by a clinical triad of microangiopathic hemolytic anemia, thrombocytopenia, and acute kidney injury. While HUS is most commonly seen in children under five years of age, particularly as a complication of gastrointestinal infections with Shiga toxin-producing Escherichia coli (STEC), its occurrence in adults is distinctly rare [3,4]. In adults, HUS typically presents as a secondary complication in the context of autoimmune disorders, malignancies, certain medications, or severe infections [5,6]. The atypical form of HUS (aHUS) results from genetic or acquired dysregulation of the complement system and shares similar clinical features [7].

HUS accounts for approximately 2%-5% of global mortality during its acute phase [7]. In selected cases of complement-mediated HUS (cHUS), eculizumab, a terminal complement inhibitor, may be considered as a treatment option [8]. Postoperative HUS following urological procedures is exceedingly rare. This case report highlights an unusual clinical course in a male patient: the development of Shiga toxin-associated HUS in a previously healthy adult shortly after HoLEP. We aim to provide a detailed account of the diagnostic challenges, clinical management, and therapeutic considerations involved in this severe and exceptionally rare complication.

## Case presentation

A 71-year-old Caucasian male with a BMI of 26.5, a prostate volume of 90 mL, a prostate-specific antigen (PSA) level of 3.6 ng/mL, and a negative digital rectal examination (DRE) was admitted for a scheduled HoLEP surgery. The main indication for the procedure was urinary retention caused by BPH, which was refractory to pharmacotherapy with tamsulosin and finasteride. The symptoms had persisted for more than three months, during which the patient had remained chronically catheterized, with two unsuccessful trials without a catheter. The patient denied the use of any other medications, reported no known allergies, and had no history of comorbidities. He did not report any symptoms of acute urinary tract infection (UTI) or gastrointestinal infection during admission. Two weeks before admission, a urine culture obtained immediately following re-catheterization had been sterile. Perioperative antibiotic prophylaxis consisted of a single dose of amikacin, per the standard protocol of our institution.

The HoLEP surgery was performed using the en-bloc technique, lasting 90 minutes, during which the whole 90 mL of prostate tissue was removed. The surgery was completed without any complications. The surgical technique, including the en-bloc enucleation and subsequent morcellation, is summarized with captions and demonstrated in Video 1. Approximately six hours after the surgery, a significantly altered mental status was observed, despite the lack of any abnormalities in laboratory tests and brain CT scan imaging. On postoperative day (PoD) one, impaired consciousness and jaundice were observed. Laboratory tests found features of acute liver failure, acute kidney injury, as well as elevated inflammation markers. Due to the suspicion of infectious multi-organ failure, empirical antibiotic therapy containing meropenem and vancomycin was initiated. The patient was transferred to the ICU.

**Video 1:** En-bloc holmium laser enucleation of a 90 mL prostate

During the ICU stay, electrolyte and coagulation disorders were corrected, and an appropriate dosage of vancomycin was established. Due to anuria and metabolic disorders, the patient underwent continuous dialysis. Blood cultures obtained upon admission to the ICU were sterile. At a follow-up visit, the patient revealed that, in the week preceding urological ward admission before HoLEP surgery, he had been in close contact with his three- and five-year-old grandchildren, who had experienced acute diarrhoea. After a six-day stay in the ICU, the patient was transferred to the nephrology department, where antibiotic therapy and renal replacement therapy (RRT) were continued using a dialysis catheter in the left femoral vein. On the eighth PoD, the left femoral vein catheter was removed, and a new one was placed in the right femoral vein. Further treatment included intermittent RRT, during which a decrease in inflammatory markers was observed. This was accompanied by an increase in diuresis, initially characterized by macroscopic hematuria, which gradually subsided.

On the 20th PoD, the patient was decatheterized, showing mild stress urinary incontinence without significant irritative lower urinary tract symptoms (LUTS) and no signs of urinary retention. Because of persistent dialysis dependence, a central line as vascular access for long-term hemodialysis was placed in the right internal jugular vein. During hospitalization in the nephrology ward, due to the patient's anemia with a hemoglobin level of 7.8 g/dL, two units of blood were transfused without any complications, resulting in an increase in hemoglobin to 9.3 g/dL. The most relevant laboratory abnormalities, along with reference values and clinical interpretation, are presented in Table 1.

**Table 1:** Summary of vital laboratory results AKI: acute kidney injury; ALT: alanine aminotransferase; AST: aspartate aminotransferase; CRP: C-reactive protein; HB: hemoglobin; INR: International normalized ratio; RBC: red blood cells; WBC: white blood cells

Parameter	Value at peak abnormality	Reference range	Comment
HB, g/dL	7.8	13.5–17.5	Severe anemia; transfused two units of RBCs
WBC count, × 10^3^/uL	24	4.0–10.0	Elevated – likely an inflammatory response
CRP, mg/L	148	<5	Very high – acute inflammatory state
Creatinine, mg/dL	4.14	0.6–1.3	Severe AKI; required dialysis
ALT , U/L	774	<45	Elevated – hepatocellular injury
AST, U/L	1075	<35	Elevated – hepatocellular injury
INR	1.3	0.8–1.2	Prolonged – coagulopathy
Platelets, × 10^3^/uL	80	150–400	Thrombocytopenia

The patient was discharged from the hospital with a referral to dialysis three times a week at a district dialysis center. Histopathological examination of the postoperative prostate tissue revealed no malignant changes. Two months after discharge from the hospital, during a follow-up hospital stay for nephrological evaluation, the patient was found to be in good general condition, exhibiting abundant, appropriate to fluid intake diuresis and not presenting any urinary incontinence. In controlled studies, the creatinine level was 2 mg/dL with a decreasing tendency. The RRT was discontinued, the central line was removed, and the patient was discharged home with a referral to the nephrology clinic for regular follow-up.

## Discussion

HoLEP is a widely used and effective procedure for treating BPH, with a relatively low incidence of major complications. The procedure significantly reduces the risk of adverse events compared to transurethral resection of the prostate (TURP) surgery, such as hyponatremia, blood transfusions, and urethral strictures [6]. Nonetheless, approximately 28% of patients still experience postoperative complications. The most frequently observed complications in the immediate postoperative period include fever (11%) and urinary retention (8%), while severe complications, such as the need for blood transfusions (2.2%) or reoperation (1.4%), are rare [1]. Over half of the complications (68%) are related to intraoperative and early postoperative events, highlighting the importance of surgical experience and the capabilities of the medical facility. HoLEP performed at high-volume centers with experienced surgeons is associated with a lower complication rate [1]. Short-term complications like irritative symptoms (9.4-58.9%) and transient stress incontinence (3.2-44%) may still occur, but most resolve within weeks. Permanent incontinence is rare, affecting only 0-2.4% of patients. Additionally, bleeding complications and the need for transfusions are uncommon, with reported rates ranging from 0.7% to 5% [2].

STEC-HUS is a thrombotic microangiopathy triggered by gastrointestinal infection with Shiga toxin-producing Escherichia coli. It is characterized by thrombocytopenia, hemolytic anaemia, and ischemic organ damage. Between 30% and 61% of patients require RRT, with an average duration of 9-10 days [4]. HUS accounts for 2%-5% of global mortality during its acute phase [7]. Treatment primarily focuses on supportive care, including fluid management, blood pressure control, and RRT, while specific therapies, such as plasma exchange and complement blockade, are still under investigation [4]. Eculizumab may also be considered as a treatment option in selected cases of cHUS [8].

Firstly, we emphasize the need for a thorough history and assessment of the risk of developing infection in patients who may have been in contact with people suffering from gastrointestinal infections during the preoperative period. In the present case, three days before admission for the planned HoLEP procedure, the patient stayed with two grandchildren, aged three and five years, who had presented symptoms of acute diarrhoea. Gastrointestinal infections in children are common and most often mild, but in the context of the immediate preoperative period, they should not be ignored. In this case, the patient's late disclosure of contact with family members who suffered from acute diarrhoea was an important factor. Such exposures, although not directly related to surgery, may contribute to rare and serious systemic complications such as HUS.

Given the temporal association between the onset of symptoms and the surgical procedure, it is important to consider the potential source of STEC infection. However, no signs of intraoperative contamination or breach in sterility were identified, and the irrigation fluids used during the HoLEP procedure were sterile and prepared per institutional protocols. Moreover, the patient reported close contact with his grandchildren, who experienced acute diarrhea in the days preceding hospital admission, which strongly supports person-to-person transmission as the most likely source of infection. This form of household transmission has been well-documented in the literature and remains a known route of STEC spread. Therefore, it is unlikely that the infection originated from surgical solutions or equipment.

Secondly, the occurrence of HUS after the HoLEP procedure raises concerns about the possibility of rare systemic complications, even with procedures generally considered safe and with a low number of complications. Urologists should remain watchful for the appearance of unusual postoperative symptoms, such as impaired consciousness, unexplained progressive multi-organ failure, and systemic inflammatory reactions. They may signal rare complications that require immediate recognition and intervention.

Finally, this report highlights the importance of long-term follow-up of patients who experience severe postoperative complications. In this case, the patient regained normal renal function and normalized diuresis. This favorable result highlights the importance of continuous nephrological evaluation and regular monitoring after hospital discharge. Long-term follow-up enables treatment adjustments, including gradual tapering of RRT and assessment of possible late complications, thereby optimizing the patient's recovery and improving long-term health outcomes.

The therapeutic success of the described case is testimony to the indispensable role of a multidisciplinary approach to patient care in complex cases. The patient's recovery was achieved thanks to close cooperation between urologists, intensive care physicians, nephrologists, and nursing staff. Early identification of multi-organ failure after HoLEP and prompt transfer to the ICU were crucial in preventing further progression of multi-organ failure. Empirical antibiotic therapy aimed at potential infectious agents was initiated, and then the nephrology team controlled the patient's acute renal failure using RRT. Communication between the various medical teams facilitated effective decision-making, which has a direct impact on the patient's recovery. An integrated approach to care emphasizes the importance of collaboration in managing rare, life-threatening complications and ensuring a comprehensive, patient-centered treatment strategy.

We believe this case report is important as it describes the first known case of HUS developing after HoLEP surgery, expanding the understanding of rare but potentially serious complications of this procedure. It contributes new insights into the diagnosis and management of HUS in the context of urological surgery, highlighting the need for careful monitoring of patients before and after procedures that are generally considered safe.

## Conclusions

This report illustrates an extremely rare but clinically significant complication following HoLEP surgery - the development of STEC-HUS in an adult patient. While the temporal association may suggest a potential link, clinical history strongly supports prior exposure to infectious agents, rather than the surgical procedure itself, as the underlying cause. Awareness of such atypical postoperative presentations is crucial, as early recognition and prompt multidisciplinary management in these rare scenarios can significantly improve outcomes in patients presenting with acute organ dysfunction following otherwise routine endourological procedures.
